# Whole‐exome sequencing provides insights into monogenic disease prevalence in Northwest Russia

**DOI:** 10.1002/mgg3.964

**Published:** 2019-09-03

**Authors:** Yury A. Barbitoff, Rostislav K. Skitchenko, Olga I. Poleshchuk, Anton E. Shikov, Elena A. Serebryakova, Yulia A. Nasykhova, Dmitrii E. Polev, Anna R. Shuvalova, Irina V. Shcherbakova, Mikhail A. Fedyakov, Oleg S. Glotov, Andrey S. Glotov, Alexander V. Predeus

**Affiliations:** ^1^ Bioinformatics Institute St. Petersburg Russia; ^2^ Department of Genetics and Biotechnology St. Petersburg State University St. Petersburg Russia; ^3^ ITMO University St. Petersburg Russia; ^4^ City Hospital No. 40 St. Petersburg Russia; ^5^ Department of Genomic Medicine D.O. Ott Research Institute of Obstetrics, Gynaecology and Reproduction St. Petersburg Russia; ^6^ Laboratory of Biobanking and Genomic Medicine of Institute of Translation Biomedicine St. Petersburg State University St. Petersburg Russia; ^7^ Cerbalab LTD St. Petersburg Russia; ^8^ Institute of Living Systems Immanuel Kant Baltic Federal University Kaliningrad Russia

**Keywords:** allele frequency, Mendelian disease, Russia, whole‐exome sequencing

## Abstract

**Background:**

Allele frequency data from large exome and genome aggregation projects such as the Genome Aggregation Database (gnomAD) are of ultimate importance to the interpretation of medical resequencing data. However, allele frequencies might significantly differ in poorly studied populations that are underrepresented in large‐scale projects, such as the Russian population.

**Methods:**

In this work, we leveraged our access to a large dataset of 694 exome samples to analyze genetic variation in the Northwest Russia. We compared the spectrum of genetic variants to the dbSNP build 151, and made estimates of ClinVar‐based autosomal recessive (AR) disease allele prevalence as compared to gnomAD r. 2.1.

**Results:**

An estimated 9.3% of discovered variants were not present in dbSNP. We report statistically significant overrepresentation of pathogenic variants for several Mendelian disorders, including phenylketonuria (PAH, rs5030858), Wilson's disease (*ATP7B*, rs76151636), factor VII deficiency (*F7*, rs36209567), kyphoscoliosis type of Ehlers‐Danlos syndrome (*FKBP14*, rs542489955), and several other recessive pathologies. We also make primary estimates of monogenic disease incidence in the population, with retinal dystrophy, cystic fibrosis, and phenylketonuria being the most frequent AR pathologies.

**Conclusion:**

Our observations demonstrate the utility of population‐specific allele frequency data to the diagnosis of monogenic disorders using high‐throughput technologies.

## 1 INTRODUCTION

Rapid development of the next‐generation sequencing (NGS) technology, including the introduction of cost‐effective targeted resequencing approaches for human genomes such as whole‐exome sequencing (WES) (Ng et al., 2009), has dramatically enhanced the applicability of NGS methods in clinical practice (Biesecker & Green, 2014). With the development of bioinformatic software for efficient and accurate data analysis, variant interpretation, i.e. classification of genetic variants by their presumed pathogenicity, has become the most challenging step on the way from raw sequence data to the definitive molecular diagnosis (Nykamp et al., 2017; Richards et al., 2015). Frequency of the non‐reference allele in the population is one of the most important factors that influence variant interpretation. Over the course of the previous decade, several large‐scale projects aimed at characterizing both global and regional frequencies of variant alleles. These included 1000 Genomes project (Auton et al., 2015) and the Exome Aggregation Consortium (ExAC)/Genome Aggregation Database (gnomAD) (Karczewski et al., 2019; Lek et al., 2016).

Despite the overwhelmingly large amount of samples in the gnomAD data (125,748 for release v. 2.1), genetic variation in many regions around the globe is still poorly studied. Many countries take efforts to fill the gap by running national genome projects (e.g., two recent studies of Qatari population [Fakhro et al., 2016; Rodriguez‐Flores et al., 2014]). One of such initiatives, the Genomes Russia project launched in 2015, aimed at characterizing the spectrum of genetic variation in diverse ethnic groups across Russia (Oleksyk, Brukhin, & O’Brien, 2015; Zhernakova et al., 2018). However, the project is currently far from being completed, and the amount of samples included into the first phase of the project is insufficient to make assumptions about the prevalence of monogenic disorders (Zhernakova et al., 2018). On the other hand, a large number of individuals have been sequenced for both clinical and research purposes in smaller projects across Russia. Previously, we have used whole exome sequencing for human genetics research (e.g., Barbitoff, Bezdvornykh, et al., 2018) and clinical practice, with the total number of exome samples analyzed reaching 694. In this work, we decided to leverage our access to this set of samples to make a first glimpse into the spectrum of genetic variation in Russia and to identify the most prevalent disease risk alleles.

## 2 MATERIALS AND METHODS

### 2.1 Sequencing data and samples

We used a set of 694 samples sequenced with both whole‐exome sequencing kits (Agilent SureSelect V6, Illumina Nextera RapidCapture, Roche SeqCap EZ MedExome, and Illumina TruSeq Exome) and clinical exome panel (CES; Illumina TruSight One sequencing kit) (105, 15.1%). This set of samples was partially described in (Barbitoff, Bezdvornykh, et al., 2018; Barbitoff, Polev, et al., 2018; Barbitoff, Serebryakova, et al., 2018). All samples were sequenced with the Illumina HiSeq 2500 and HiSeq 4000 sequencing instruments. The dataset contained individuals aggregated from different research and clinical projects (both controls and individuals with disease [major phenotypes: maturity onset diabetes of the young (MODY), type 2 diabetes (T2D), obesity, autistic spectrum disorders (ASD), connective tissue disorders (CTD), and neurofibromatosis]) most of the individuals were of Russian (~80%) ethnicity (self‐reported) and Caucasian race; with the study participants predominantly being residents of the North‐Western region of Russia.

### 2.2 Bioinformatic analysis

Bioinformatic analysis of exome sequencing data was performed using a custom pipeline based on the bwa aligner (Li & Durbin, 2009), Genome Analysis Toolkit v. 3.5., and Picard tools v. 2.2.2. The pipeline was constructed according to the GATK Best Practices workflow (DePristo et al., 2011; van der Auwera et al., 2013). All samples were processed with identical pipeline settings and genotyped jointly using the cohort genotyping method in GATK. Variants were annotated with SnpEff/SnpSift (Cingolani et al., 2012) using the following resources: 1000 Genomes project allele frequencies (Auton et al., 2015), gnomAD r.2.1 allele frequencies (Karczewski et al., 2019; Lek et al., 2016), ESP6500 allele frequencies (Fu et al., 2013), pathogenicity predictions from dbNSFP (Liu et al., 2016), ClinVar database v. 2019‐01‐10.

### 2.3 Statistical assessment of the variants

To analyze the spectrum of genetic variants in the dataset, we used all SNP calls from all samples. To compare the variant call qualities of known and novel variant sites, we fitted a linear regression model to predict site‐level quality score based on variant type (known/novel) and alternative allele frequency. Statistical significance of the model coefficients was assessed using the *t*‐statistic. To evaluate the prevalence of disease alleles, we narrowed the dataset down to a set of individuals with well‐established phenotype without severe early‐onset Mendelian pathologies (the resulting dataset comprised population controls, individuals with multifactorial obesity, MODY, T2D, ASD, and mild forms of CTD). All samples that were reported as relatives, as well as all individual that share an unusually high proportion of heterozygous calls (as reported by the QC3 package (Guo et al., 2014) were removed to avoid inclusion of related samples that were potentially mislabeled (Supporting Information Figure S1). We focused on known pathogenic variants reported in ClinVar, and limited the analysis to genes that are linked to Mendelian disorders with autosomal recessive inheritance pattern. To enforce the analysis of allele prevalence, we obtained a binomial *p*‐value of observing *n* alleles in the data is sampling *N* chromosomes from the population with true alternative allele frequency equal to gnomAD AF for either whole dataset or for the non‐Finnish European (NFE) individuals only. We manually curated all of the findings to ensure that disease allele carriers are not parts of trios of individuals carrying the disease. Expected disease incidence was calculated under the assumption of Hardy‐Weinberg equilibrium as the square of cumulative frequency of all pathogenic variants found in a certain gene.

### 2.4 Data availability

All scripts pertinent to the analysis are available through GitHub: https://github.com/bioinf/afpaper. Per gene disease variant counts complemented with the corresponding disease frequency estimates can be found in Supplementary Information Table S1. To access the VCF file containing allele frequency information please contact the authors.

## 3 RESULTS

### 3.1 The spectrum of identified exome variants

**Figure 1:**
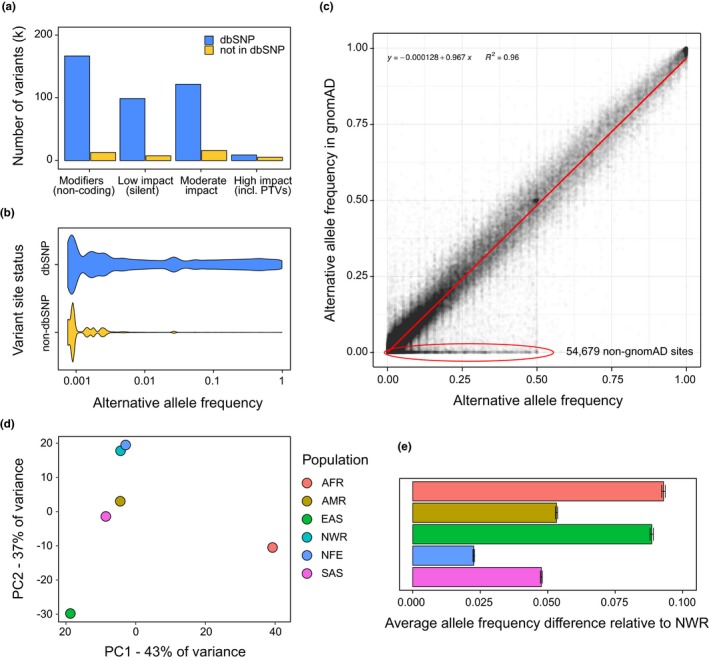
The spectrum of genetic variation in exomes of Northwest Russia. (a) Distribution of the identified variant sites by functional impact on protein structure as given by SnpEff. (b) Alternative allele frequency distribution for known (dbSNP) and novel (non‐dbSNP) variants. (c) A scatterplot of alternative allele frequencies in the dataset versus gnomAD‐based frequencies; gnomAD‐only sites, multiallelic entries, and poorly covered regions are excluded. Red circle highlights sites not present in gnomAD. (d) Principal component analysis of allele frequencies for 121,171 exome variants present in Northwest Russia and all of the gnomAD populations. AFR, African; AMR, Ad Mixed American; EAS, East Asian; NFE, non‐Finnish European; NWR, Northwest Russia; SAS, South Asian. (e) Root mean square difference in allele frequencies between NWR and different gnomAD populations for the same variant sites as in (d). Error bars represent the 95% confidence interval margins for the mean

We first set out to characterize the spectrum of genetic variants found in our sample. For the complete set of 694 study participants, we identified a total of 463,100 variant sites inside targeted exome regions. Out of these, 420,187 (90.7%) had an rsID according to the latest dbSNP build 151, and the remaining 42,913 variants were not previously reported. To ensure these non‐dbSNP variants are not predominantly composed of variant calling artifacts, we compared the distribution of quality scores for known and novel variants and found no evident differences, with novel sites having even slightly higher average quality score (linear regression coefficient *p*‐value = 0.034, Supplementary Information Figure S2, see Materials and Methods). We also observed no substantial differences in the total depth or mapping quality between dbSNP and non‐dbSNP variant sites, as well as only slight decrease in the variant allele frequency for heterozygous genotypes at novel sites (Supplementary Information Figure S3). We then assessed the distribution of these variant sites across functional impact categories as classified by SnpEff (Cingolani et al., 2012). As expected, a vast majority of variants were silent or non‐coding variants (300,117, 64.8%); 14,106 variants were classified as high‐impact variants (with the majority of sites corresponding to protein‐truncating variants), with an average of 622.5 high‐impact variants per WES sample (134 for CES; per‐sample statistics are shown on Supporting Information Figure S4). Interestingly, a large fraction of the variants that belonged to the high‐impact group were not reported in dbSNP (5143/14106, chi‐squared p‐value with respect to the overall distribution <2.2 × 10^–16^). This is concordant with the observation that most novel variants identified had lower alternative allele frequency (Figure 1b, Supporting Information Figure S5). We then went on to assess the concordance between alternative allele frequency in our sample and in the gnomAD r. 2.1 exomes. To this end, we narrowed down our dataset to sites that have mean coverage of at least 15x across gnomAD exomes, and then assessed the linear regression coefficients for the allele frequencies. We found strong correlation between AFs within gnomAD and our dataset (*R* ^2^ = 0.96, Figure 1c, Supporting Information Figure S6). At the same time, a total of 54,679 variants have not been reported in gnomAD. Most of these non‐gnomAD variants were also missing from dbSNP build v. 151 (37,338, 68.2%), indicating that these variants constitute a specific component of the genetic structure of the population of Northwest Russia.

We then investigated the relationship between the Northwest Russia (NWR) population and the major populations present in gnomAD. We first examined the correlation between alternative allele frequencies in NWR and in five major ancestral groups: African (AFR), Ad Mixed American (AMR), East Asian (EAS), non‐Finnish European (NFE), and South Asian (SAS). Expectedly, we observed the highest concordance of NWR allele frequencies with the AFs derived from the NFE population (Supporting Information Figure S7). Principal component analysis of allele frequencies across 121,171 exome variant sites present in our dataset and in all five gnomAD populations also showed that the NWR populations falls in close proximity to the non‐Finnish Europeans (Figure 1d). To further validate these observations, we also calculated the root‐mean‐square deviation in allele frequencies between our dataset and gnomAD populations. Concordantly with the aforementioned findings, we observed the lowest average difference for the NFE populations (Figure 1e). These results are in good concordance with the primary data of the Genomes Russia project (Zhernakova et al., 2018).

### 3.2 Analysis of disease allele prevalence

We then moved on to the assessment of the prevalence of disease‐causing alleles in our sample. Firstly, we have further narrowed the dataset down to a set of individuals that have a well‐established phenotype without severe early‐onset disease. Such filtering resulted in a set of 372 unrelated individuals which contained 314,902 of 463,100 (68.0%) variant calls. We then focused on variants that are reported as pathogenic in ClinVar, have a relatively low (<0.5%) frequency in gnomAD, and are strictly heterozygous in the curated dataset. To statistically enhance our analysis of the allele prevalence, we calculated the binomial p‐value of obtaining the observed alternative allele counts if the true population AF matched the one reported for the NFE population in gnomAD (see Materials and Methods).

We found several notable examples of highly prevalent known pathogenic variants for autosomal recessive disorders in our sample (summarized in Table 1). Of highest frequency was the rs5030858 variant in the *PAH* gene (MIM#612349; gnomAD NFE AF = 0.0015, *p* = 7.9 × 10^–4^), a well‐established and one of the most frequent pathogenic variant for phenylketonuria (Tighe et al., 2003). Allele carriers for this variant were observed 6 times in smaller and 11 times in larger dataset, which corresponds to an estimated allele frequency of 0.0081. The second prevalent disease allele in the dataset was the rs36209567 variant in the factor VII (*F7,* MIM#613878) gene (gnomAD NFE AF = 0.001, *p* = .001), a mutation responsible for factor VII deficiency, a disorder that is extremely rare in general population (1:500,000, according to Wulff & Herrmann, 2000). Another example of a highly prevalent disease variant in our dataset was the rs61754365 variant in the *TYR* gene (MIM#606933) linked to tyrosinase‐negative albinism (Takeda, Tomita, Matsunaga, Tagami, & Shubahar, 1990) (gnomAD NFE AF = 3.2 × 10^–4^, *p* = 1.1 × 10^–4^). Of interest is the higher incidence of a pathogenic rs76151636 variant (gnomAD NFE AF = 0.0013, *p* = .016) in the *ATP7B* gene (MIM#606882), the causal gene for Wilson's disease (WD). The H1069Q mutation corresponding to rs76151636 is reported as the most prevalent WD mutation in Europe and North America (de Bie, Muller, Wijmenga, & Klomp, 2007), which is consistent with our findings. We also observed unusually high prevalence of the rs542489955 variant in the *FKBP14* gene (MIM#614505; gnomAD NFE AF = 0.001, *p* = .0061), a frameshift mutation linked to the kyphoscoliosis type of Ehlers‐Danlos syndrome (EDS) (Baumann et al., 2012). This variant is the most frequent EDS mutation in our dataset, which is unusual given that *FKBP14*‐related EDS appears to be one of the rare forms of the disease. Among the less prevalent pathogenic variants, we observed statistically significant overrepresentation of disease alleles for Charcot‐Marie‐Tooth disease type 4B3 (*SBF1*, MIM#603560; rs200488568, *p* = 1.2 × 10^–4^); Maple syrup urine disease (*BCKDHB*, MIM#248611; rs386834233, *p* = .003), combined oxidative phosphorylation deficiency 10 (*MTO1*, MIM#614667; rs201544686, *p* = 4.9 × 10^–4^) (3 observations each), and several other recessive pathologies. Notably, we identified no prevalent protein‐truncating variants missing from ClinVar (in AR disease‐related genes), suggesting that the majority of disease alleles are shared between Russian and European populations. We also analyzed the frequency of each identified prevalent disease allele in gnomAD populations other than NFE. Interestingly, we found that one of the variants, rs38683423 in *BCKDHB*, is also overrepresented in the Finnish population, possibly indicating either gene flow between NWR and Finnish population or region‐specific selection against the related condition.

**Table 1:** Prevalent disease alleles in the Northwest Russia dataset

Location	rsID	Gene	gnomAD AF	gnomAD NFE AF	Allele count^a^	Estimated AF (lower/upper CI)^b^	*p*‐value^a^	Disease/condition
12:103234271	rs5030858	*PAH*	7.6 × 10^−4^	0.0015	6 (11)	0.0081 (0.0037/0.0175)	7.9 × 10^−4^ (1.1 × 10^−5^)	Phenylketonuria
13:113772982	rs36209567	*F7*	5.6 × 10^−4^	0.0010	5 (7)	0.0067 (0.0029/0.0157)	0.0010 (5.7 × 10^−4^)	Factor VII deficiency
7:30058726	rs542489955	*FKBP14*	5.5 × 10^−4^	0.0010	4 (8)	0.0054 (0.0021/0.0138)	0.0061 (9.6 × 10^−5^)	Ehlers‐Danlos syndrome, kyphoscoliotic type, 2
11:88911771	rs61754365	*TYR*	2.9 × 10^−4^	3.2 × 10^−4^	4 (8)	0.0054 (0.0032/0.0139)	1.1 × 10^−4^ (2.4 × 10^−8^)	Tyrosinase‐negative oculocutaneous albinism
13:52518281	rs76151636	*ATP7B*	9.2 × 10^−4^	0.0013	4 (6)	0.0053 (0.0021/0.0138)	0.0159 (0.0095)	WIlson's disease
22:50893287	rs200488568	*SBF1*	3.3 × 10^−4^	1.4 × 10^−4^	3 (4)	0.0045 (0.0015/0.0133)	1.2 × 10^−4^ (5.0 × 10^−5^)	Charcot‐Marit‐Tooth disease, type 4B3
2:152357937	rs549794342	*NEB*	2.7 × 10^−4^	4.7 × 10^−4^	3 (6)	0.0040 (0.0014/0.0118)	0.0050 (5.9 × 10^−5^)	Nemaline myopathy
6:74191932	rs201544686	*MTO1*	1.7 × 10^−4^	2.0 × 10^−4^	3 (5)	0.0040 (0.0014/0.0118)	4.9 × 10^−4^ (5.4 × 10^−6^)	Combined ox. phos. deficiency 10
6:80910740	rs386834233	*BCKDHB*	5.5 × 10^−4^	3.9 × 10^−4^	3 (4)	0.0040 (0.0014/0.0118)	3.0 × 10^−3^ (2.3 × 10^−3^)	Maple syrup urine disease
5:54527618	rs775051461	*CCNO*	9.8 × 10^−5^	4.7 × 10^−5^	2 (2)	0.0030 (8.3 × 10^−4^/0.0110)	4.6 × 10^−4^ (0.0015)	Ciliary dyskinesia
17:8015495	rs121434233	*ALOXE3*	1.5 × 10^−4^	2.8 × 10^−4^	2 (5)	0.0027 (7.4 × 10^−4^/0.0098)	0.018 (5.2 × 10^−5^)	Autosomal recessive congenital ichthyosis 3
18:21119369	rs543206298	*NPC1*	7.6 × 10^−5^	1.1 × 10^−4^	2 (3)	0.0027 (7.4 × 10^−4^/0.0098)	0.0033 (5.2 × 10^−4^)	Niemann‐Pick disease
8:75276240	rs104894080	*GDAP1*	3.2 × 10^−5^	7 × 10^−5^	2 (2)	0.0027 (7.4 × 10^−4^/0.0097)	0.0013 (0.0044)	Polyneuropathy, Charcot‐Marie‐Tooth intermediate A
2:73677806	rs1307458231	*ALMS1*	2.0 × 10^−5^	4.4 × 10^−5^	2 (2)	0.0027 (7.4 × 10^−4^/0.0097)	5.0 × 10^−4^ (1.8 × 10^−3^)	Alstrom syndrome

a Values in brackets are derived from larger uncurated sample (see Materials and Methods). *p*‐values are calculated using allele frequencies in the gnomAD non‐Finnish European (NFE) population.

b 95% exact confidence interval margins for binomial proportions are given.

We then went on to estimate the prevalence of monogenic disorders based on the cumulative frequencies of pathogenic variants for these diseases. To this end, we obtained per‐gene counts of all pathogenic alleles in both curated and larger uncurated dataset (Supplementary Information Table S1). We focused on disease with more than 3 observations of reported pathogenic alleles in the curated dataset. Results of the prevalence analysis are summarized in Table 2. 12 genes passed our filtering criteria, with the following disorders being the most prevalent: (a) Stargardt disease (with *ABCA4,* MIM#601691, as the major gene, as also reported previously (Sheremet et al., 2017), incidence at least 1:3226), (b) cystic fibrosis (*CFTR*, MIM#602421, with the F508del (7:117199644:ATCT>A) mutation being the dominant variant), estimated incidence 1:5263, (c) phenylketonuria (*PAH* gene, incidence of up to 1:5556), (d) hepatic lipase deficiency (*LIPC*, MIM#151670, 1:10000, with one pathogenic variant rs113298164), and (e) tyrosinase‐negative oculocutaneous albinism (*TYR*, 1:13158). Remarkably, our estimates of cystic fibrosis, galactosemia, and phenylketonuria incidence were concordant with previous gene‐level estimates (Abramov, Kadochnikova, Yakimova, Belousova, Maerle, Sergeev, Ragimov, Donnikov, & Trofimov, 2015, Abramov, Belousova, Kadochnikova, Ragimov, & Trofimov, 2017). On the other hand, estimated incidences of factor VII deficiency and congenital afibrinogenemia were approximately 20 to 100 times higher than the reported global ones (Mannucci, Duga, & Peyvandi, 2004; Wulff et al., 2000), though the sample size limitation did not allow us to reliably estimate the degree of this discordance.

**Table 2:** Disease prevalence estimated from known pathogenic variants’ frequencies

Disease/condition	Gene(s)	Allele count^a^	Carrier frequency (lower/upper CI)	Disease frequency (lower/upper CI)^a^	Known frequency^b^	Comments/ references
Retinal dystrophy, Stargardt disease	*ABCA4*	13 (23)	0.0350 (0.0206/0.0589)	3.1 × 10^−4^ (1.1 × 10^−4^/8.8 × 10^−4^)	1 in 10,000	Zol’nikova, 2016; Sheremet et al., 2017
Cystic fibrosis	*CFTR*	11 (19)	0.0296 (0.0167/0.0522)	2.2 × 10^−4^ (6.9 × 10^−5^/6.9 × 10^−4^)	1 in 10,000	Reported carrier frequency of 0.032 (Abramov et al., 2015)
Phenylketonuria	*PAH*	11 (18)	0.0296 (0.0167/0.0522)	2.2 × 10^−4^ (6.9 × 10^−5^/6.9 × 10^−4^)	1 in 10,000	Reported carrier frequency of 0.029 (Abramov et al., 2015)
Afibrinogenemia, congenital	*FGG*	7 (10)	0.0190 (0.0092/0.0387)	9.0 × 10^−5^ (2.1 × 10^−5^/3.8 × 10^−4^)	n.a.	Reported lobal frequency of 1 in 1,000,000 (Mannucci et al., 2004)
Hepatic lipase deficiency	*LIPC*	6 (14)	0.0162 (0.0075/0.0359)	6.6 × 10^−5^ (1.4 × 10^−5^/3.1 × 10^−4^)	n.a.	—
Tyrosinase‐negative oculocutaneous albinism	*TYR*	6 (12)	0.0162 (0.0075/0.0359)	6.6 × 10^−5^ (1.4 × 10^−5^/3.1 × 10^−4^)	1 in 39,000	—
Peeling skin syndrome	*TGM5*	5 (8)	0.0135 (0.0058/0.0311)	4.5 × 10^−5^ (8.3 × 10^−6^/2.5 × 10^−4^)	n.a.	—
Factor VII deficiency	*F7*	5 (7)	0.0135 (0.0058/0.0311)	4.6 × 10^−5^ (8.3 × 10^−6^/2.5 × 10^−4^)	n.a.	Reported global frequency of 1 in 500,000 (Wulff *et al.*, 2000)
Wilson's disease	*ATP7B*	4 (6)	0.0108 (0.0042/0.0274)	2.9 × 10^−5^ (4.3 × 10^−6^/1.9 × 10^−4^)	1 in 30,000.	Similar global incidence reported (Ala, Walker, Ashkan, Dooley, & Schilsky, 2007)
Ehlers‐Danlos syndrome, kyphoscoliotic type, 2	*FKBP14*	4 (8)	0.0108 (0.0042/0.0274)	2.9 × 10^−5^ (4.3 × 10^−6^/1.9 × 10^−4^)	n.a.	—
Fructose intolerance, hereditary	*ALDOB*	4 (7)	0.0108 (0.0042/0.0274)	2.9 × 10^−5^ (4.3 × 10^−6^/1.9 × 10^−4^)	n.a.	—
Galactosemia	*GALT*	4 (5)	0.0108 (0.0042/0.0274)	2.9 × 10^−5^ (4.3 × 10^−6^/1.9 × 10^−4^)	1 in 20,000	Reported carrier frequency of 0.006 (Abramov et al., 2015)

a Values in brackets are derived from larger uncurated sample (see Materials and Methods).

b Frequency data for Russian population were obtained from literature and/or clinical genetics laboratories. n.a., no data available.

## 4 DISCUSSION

The genetic structure of human populations is being extensively studied all around the world. Lots of ongoing large‐scale genome projects aim at characterizing the variants that persist in each country or region, e.g. Genomics England (Walter et al., 2015). However, there still are many white spots on the genetic map of the world which will hardly be filled in the upcoming years. With the Genomes Russia project (Oleksyk et al., 2015) being yet far from completion, Russia remains one of such white spots. To fill this gap and provide primary data on disease allele prevalence in the North‐Western region of Russia, we utilized a dataset of 694 whole‐exome and clinical exome samples sequenced for the purposes of molecular diagnostics and/or scientific projects in Saint‐Petersburg.

Sets of samples that contain diseased and related individuals should be treated with caution and cannot be used as a confident source of true population allele frequency. To avoid potential false discoveries, we applied a multi‐step curation strategy to identify prevalent disease alleles and make primary estimates of disease incidence. The data curation procedure included both exclusion of individuals with poor phenotypic description and/or severe early‐onset Mendelian disorders and accurate confirmation of non‐diseased phenotype at the validation stage.

We demonstrate that a large portion of genetic variation in Northwest Russia is specific to this particular region, with 9.3% of variant sites identified across our dataset being not reported in the latest dbSNP build 151. Notably, we observed a generally high correspondence between the allele frequencies for exome variants between Northwest Russia and the non‐Finnish European population from gnomAD (Figure 1). This findings supports the need for a large‐scale national genetic variation database in Russia, which would support both local and global clinical genetics research. We also show that many of the previously reported pathogenic alleles that are highly prevalent in European population are also overrepresented in residents of Northwest Russia (Table 1), with the allele frequencies for many these alleles in Russia being substantially higher than in non‐Finnish Europeans. These include a dominant Wilson's disease allele (rs76151636, de Bie et al., 2007), and the common phenylketonuria R408W (rs5030858) mutation in the *PAH* gene (Tighe et al., 2003). Moreover, we identified no highly prevalent pathogenic or likely pathogenic variants missing from ClinVar or dbSNP in the AR disease‐related genes, indicating that, at least for recessive pathologies, much of the genetic determinants are shared between Russia and other populations.

Our dataset allowed us to make the first exome‐level estimates of prevalence of monogenic disorders in the region (Table 2). Despite the low precision of the estimates due to the limited sample size, our data on two of the most common pathologies, cystic fibrosis and phenylketonuria, are concordant with previous gene‐level estimates. On the other hand, we highlight many pathologies which are at relatively high risk in Russia, including Wilson's disease, factor VII deficiency, Stargardt disease, tyrosinase‐negative oculocutaneous albinism, and several other diseases. Our results are also concordant with previous large‐scale analyses of disease allele carrier frequency for cystic fibrosis for individuals of Caucasian race (Lazarin et al., 2013). Moreover, estimated incidence of phenylketonuria in Russia is also similar to the one reported in diverse human populations, including a recent study in China (Zhao et al., 2019).

Overall, our results demonstrate the urgent need for population‐specific genetic databases for variant interpretation purposes and identification of disease risk factors in poorly studied populations. Larger sample sizes are certainly required to make confident assumptions about the prevalence of monogenic disorders and population frequencies of disease alleles. Nevertheless, we are hopeful that the data presented would assist medical genetics studies and clinical genetic analyses both inside and outside Russia.

## CONFLICT OF INTEREST

The authors declare no conflict of interest.

## ETHICAL COMPLIANCE

All individual studies unified here were approved by the ethical committee of the Biobank research center, and all participants provided written informed consent.
