# Supplementary material for: Whole‐exome sequencing provides insights into monogenic disease prevalence in Northwest Russia
Source: Mol Genet Genomic Med. 2019 Sep 3;7(11):e964. doi: 10.1002/mgg3.964 (PMC6825859; doi:10.1002/mgg3.964)
Supplement: Supplementary file 1 [file MGG3-7-e964-s001.pdf]

Whole-exome sequencing provides insights into monogenic disease prevalence in Northwest Russia

Yury A. Barbitoff[1,2]*, Rostislav K. Skitchenko[1,3]*, Olga I. Poleshchuk[1], Anton E. Shikov[1,4], Elena A. Serebryakova[5], Yulia A. Nasykhova[5,6], Dmitrii E. Polev[7], Anna R. Shuvalova[7], Irina V. Shcherbakova[5], Mikhail A. Fedyakov[4], Oleg S. Glotov[4,5], Andrey S. Glotov[4,5,6,8], Alexander V. Predeus[1]

1 - Bioinformatics Institute, St. Petersburg, Russia

2 - Dpt. of Genetics and Biotechnology, St. Petersburg State University, St. Petersburg, Russia

3 - ITMO University, St. Petersburg, Russia

4 - City Hospital No. 40, St. Petersburg, Russia

5 - Laboratory of Prenatal Diagnostics of Hereditary Diseases, FSBSI «The Research Institute of Obstetrics, Gynaecology and Reproductology Named after D.O. Ott», St. Petersburg, Russia

6 - Laboratory of Biobanking and Genomic Medicine of Institute of Translation Biomedicine, St. Petersburg State University, St. Petersburg, Russia

7 - Serbalab LTD, St. Petersburg, Russia

8 - Institute of Living Systems, Immanuel Kant Baltic Federal University, Kaliningrad, Russia

* - these authors contributed equally to the work

Correspondence should be addressed to: Alexander V. Predeus, predeus@bioinf.me

Keywords: whole-exome sequencing, allele frequency, Mendelian disease, Russia

SUPPLEMENTARY INFORMATION

CONTENTS

SUPPLEMENTARY FIGURES.. 2

SUPPLEMENTARY FIGURES

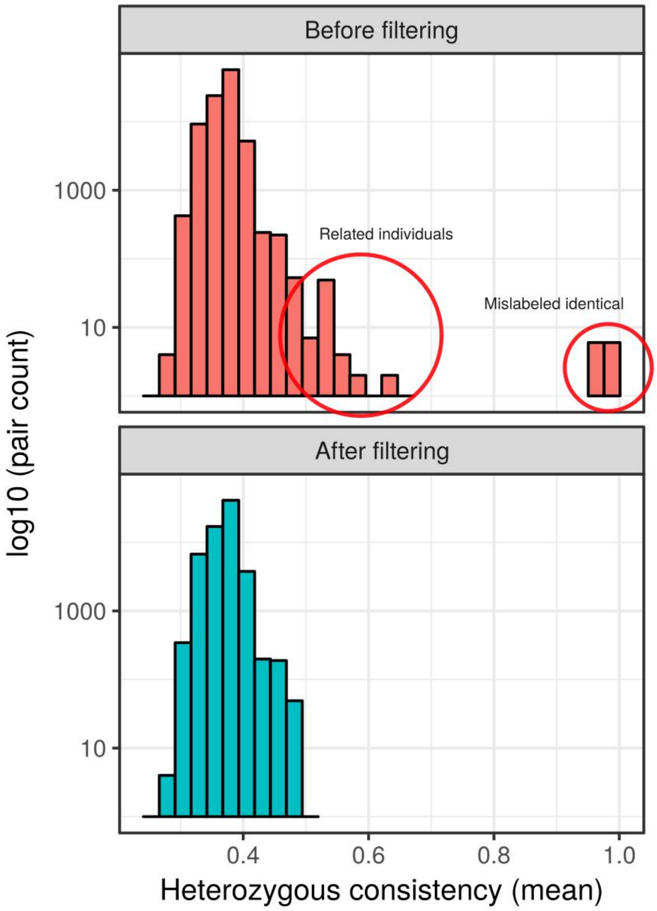

Figure S1. Consistency of heterozygous genotypes between pairs of samples before (top) and after (bottom) exclusion of known relatives and additional filtering based on heterozygous consistency (thresholded at 0.5). Values were calculated with the QC3 package (Guo et al., 2014).

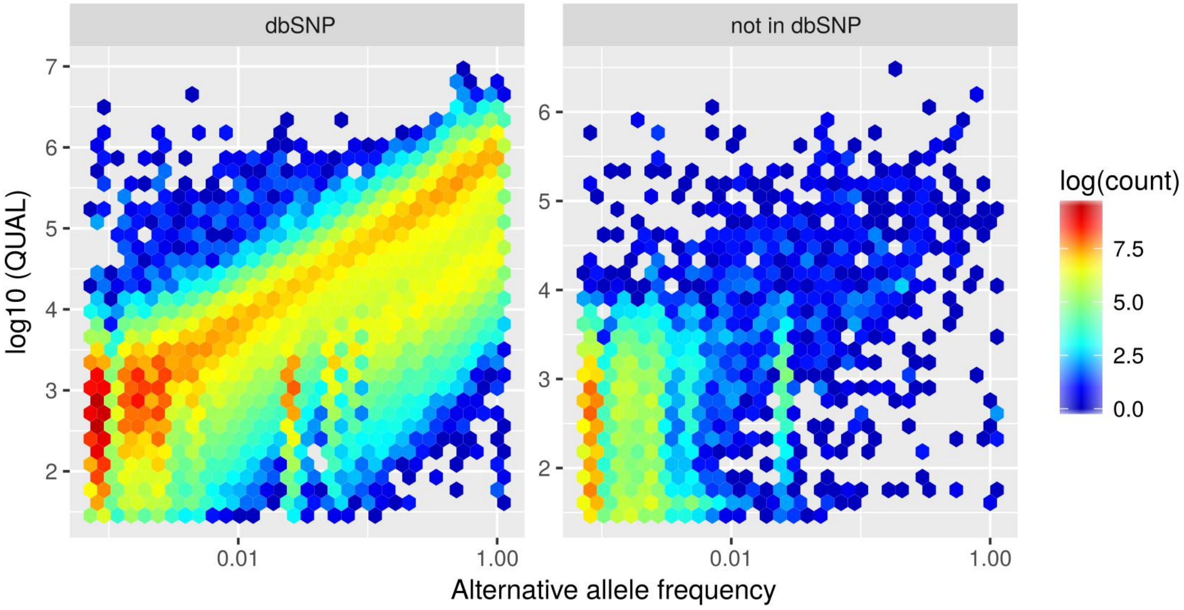

Figure S2. Comparison of the quality score distributions for variants reported or not reported in dbSNP build 151. Hexagon color represents count of variant sites in logarithmic scale.

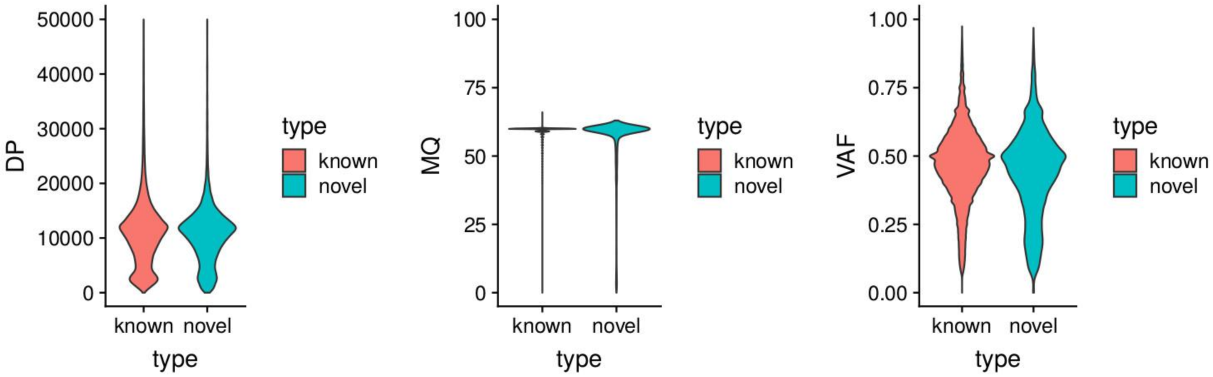

Figure S3. Distributions of site-level depth (DP), mapping quality (MQ), and heterozygous genotype variant allele frequencies (VAF) at known (dbSNP, red) and novel (not in dbSNP, blue) variant sites.

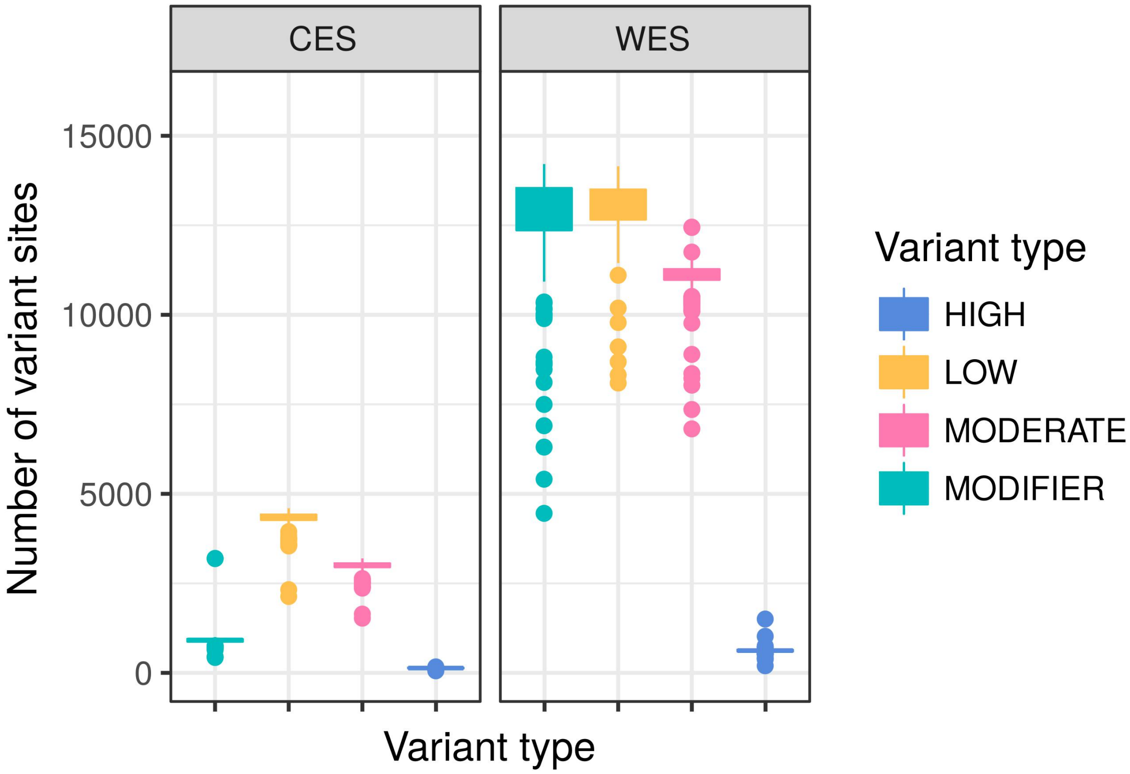

Figure S4. Distribution of the numbers of varinats called in each whole-exome (WES, right) and Illumina TruSight One (CES, left) sample separated by variant effect type (as provided by SnpEff annotation (Cingolani et al., 2012).

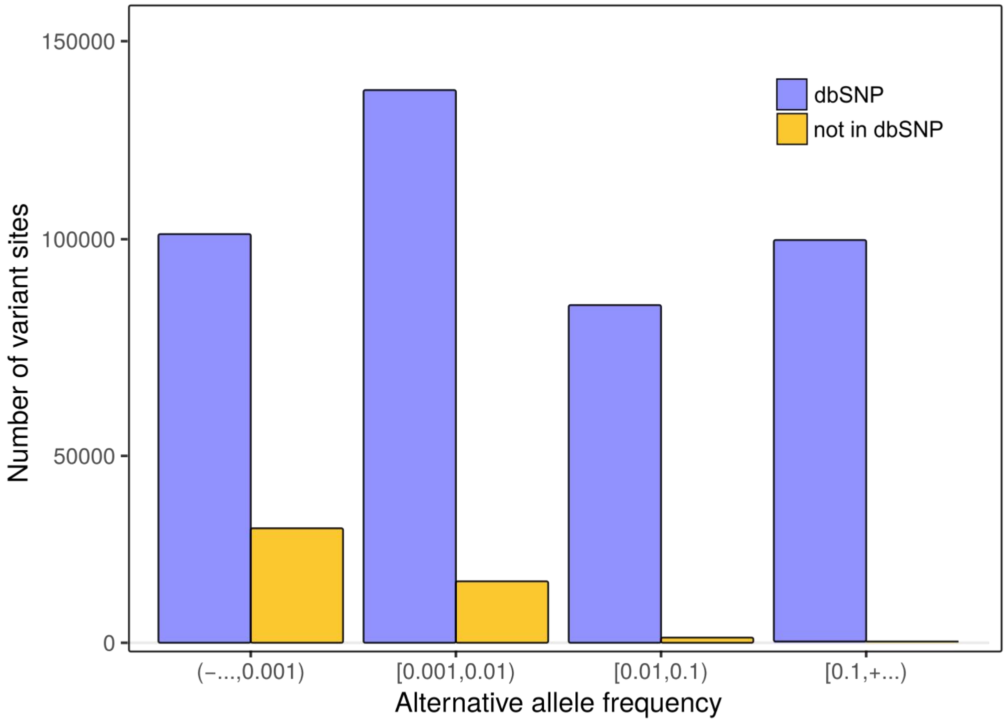

Figure S5. Proportions of dbSNP and non-dbSNP sites in the dataset depending on the frequency of alternative allele. Note that the vast majority of novel variants have an AF < 0.01.

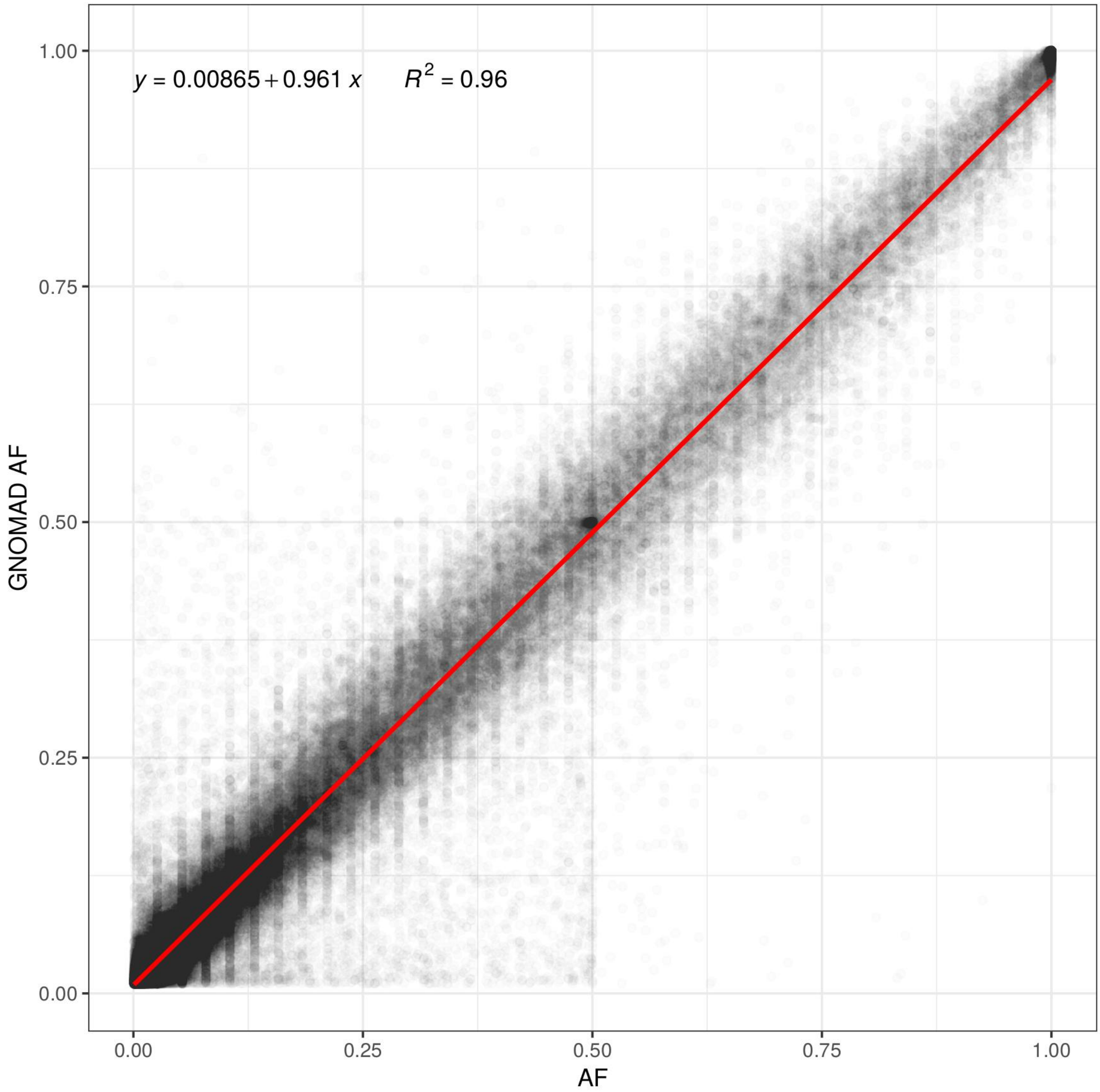

Figure S6. Scatterplot of alternative allele frequencies in gnomAD vs. North-Western Russia dataset (with primary filtering for variants that have AF >= 0.01 in gnomAD, *i.e.* NWR-specific sites are removed).

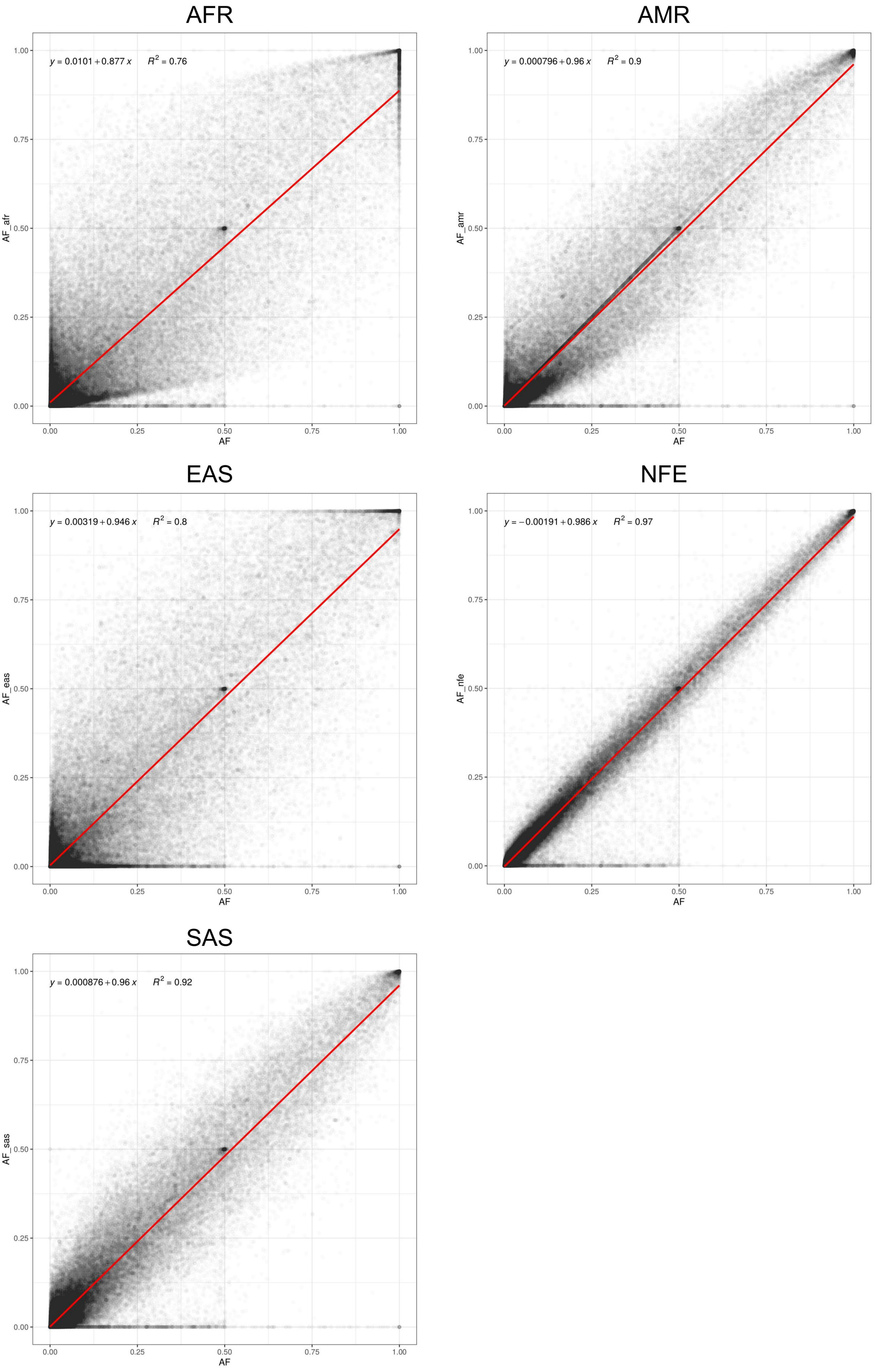

Figure S7. Scatterplot of alternative allele frequencies for different gnomAD populations (AFR, African; AMR, Americam; EAS - East Asian; NFE - non-Finnish European; SAS - South Asian) vs. North-Western Russia dataset.
